# Molecular subtypes based on DNA sensors predict prognosis and tumor immunophenotype in hepatocellular carcinoma

**DOI:** 10.18632/aging.204870

**Published:** 2023-07-14

**Authors:** Hong-Sheng Lin, Wen-Peng Pang, Hao Yuan, Yin-Zhi Kong, Fu-Li Long, Rong-Zhen Zhang, Li Yang, Qiao-Ling Fang, Ai-Ping Pan, Xiao-Hui Fan, Ming-Fen Li

**Affiliations:** 1 Department of Microbiology, School of Basic Medical Sciences, Guangxi Medical University, Nanning 530021, China; 2 Department of Clinical Laboratory, The First Affiliated Hospital of Guangxi University of Chinese Medicine, Nanning 530023, China; 3 Department of Immunology, School of Basic Medical Sciences, Guangxi Medical University, Nanning 530021, China; 4 Department of Hepatology, The First Affiliated Hospital of Guangxi University of Chinese Medicine, Nanning 530023, China

**Keywords:** hepatocellular carcinoma, DNA sensors, immune microenvironment, prognosis, immunotherapy

## Abstract

DNA sensors play crucial roles in inflammation and have been indicated to be involved in antitumor or tumorigenesis, while it is still unclear whether DNA sensors have potential roles in the prognosis and immunotherapy of hepatocellular carcinoma (HCC). Herein, The Cancer Genome Atlas and Gene Expression Omnibus databases were used to analyze RNA sequencing data and clinical information. A total of 14 DNA sensors were collected and performed consensus clustering to determine their molecular mechanisms in HCC. Two distinct molecular subtypes (Clusters C1 and C2) were identified and were associated with different overall survival (OS). Immune subtype analysis revealed that C1 was mainly characterized by inflammation, while C2 was characterized by lymphocyte depletion. Immune scoring and immunomodulatory function analysis confirmed the different immune microenvironment of C1 and C2. Notably, significant differences in “Hot Tumor” Immunophenotype were observed between the two subtypes. Moreover, the prognostic model based on DNA sensors is capable of effectively predicting the OS of HCC patients. Besides, the chemotherapeutic drug analysis showed the different sensitivity of two subtypes. Taken together, our study shows that the proposed DNA sensors were a reliable signature to predict the prognosis and immunotherapy response with potential application in the clinical decision and treatment of HCC.

## INTRODUCTION

Primary liver cancer is the sixth most common cancer and the third leading cause of cancer-related death worldwide [1]. Pathologically, there are three categories: hepatocellular carcinoma (HCC) (75-85%), intrahepatic cholangiocarcinoma (10-15%), and some other rare types [2]. At present, multiple therapeutic strategies are used in HCC treatment, such as surgery, tumor ablation, transarterial treatment, and systemic therapy [3]. It is notable that systemic therapy is indispensable in approximately 50-60% patients [3]. In recent years, immune checkpoint inhibitors (ICIs)-based immunotherapy has shown significant efficacy in systemic therapy for HCC. HCC patients can gain more benefits from ICIs in terms of their long-term survival, tumor response rate, and the duration of response [4]. Unfortunately, only 15-20% HCC patients are responsive to ICIs [5]. Therefore, there is a critical need to identify biomarkers capable of predicting ICIs response and improving the response of HCC patients to ICIs therapy.

DNA defective mismatch repair (dMMR) and microsatellite instability (MSI) that are driven by gene mutations can induce DNA damage response (DDR) in tumor cells [6]. Pan-cancer analysis suggested that DDR improved the response of HCC patients to ICIs therapy. DDR, therefore, is considered a promising target for improving tumor response to immunotherapy [7, 8]. There are three steps that compose DDR: cytoplasmic transfer of host cellular DNA; cytoplasmic DNA (cytoDNA) sensing; a series of signal transductions that activate innate immune response and then induce cellular senescence and death [9]. Amongst the three steps, cytoDNA sensing is particularly important [10]. CytoDNA sensing is emerging as key mediators of inflammation in diverse physiological and pathological contexts which can drive inflammation-related cytokines in the absence of infection [11, 12]. Besides, cytoDNA sensing by DNA sensors is intimately connected to the secretion of cytokines that support innate and adaptive antitumor immunity [11]. However, the specific role and molecular mechanism of DNA sensors in HCC remain to be open issues.

**Figure 1:**
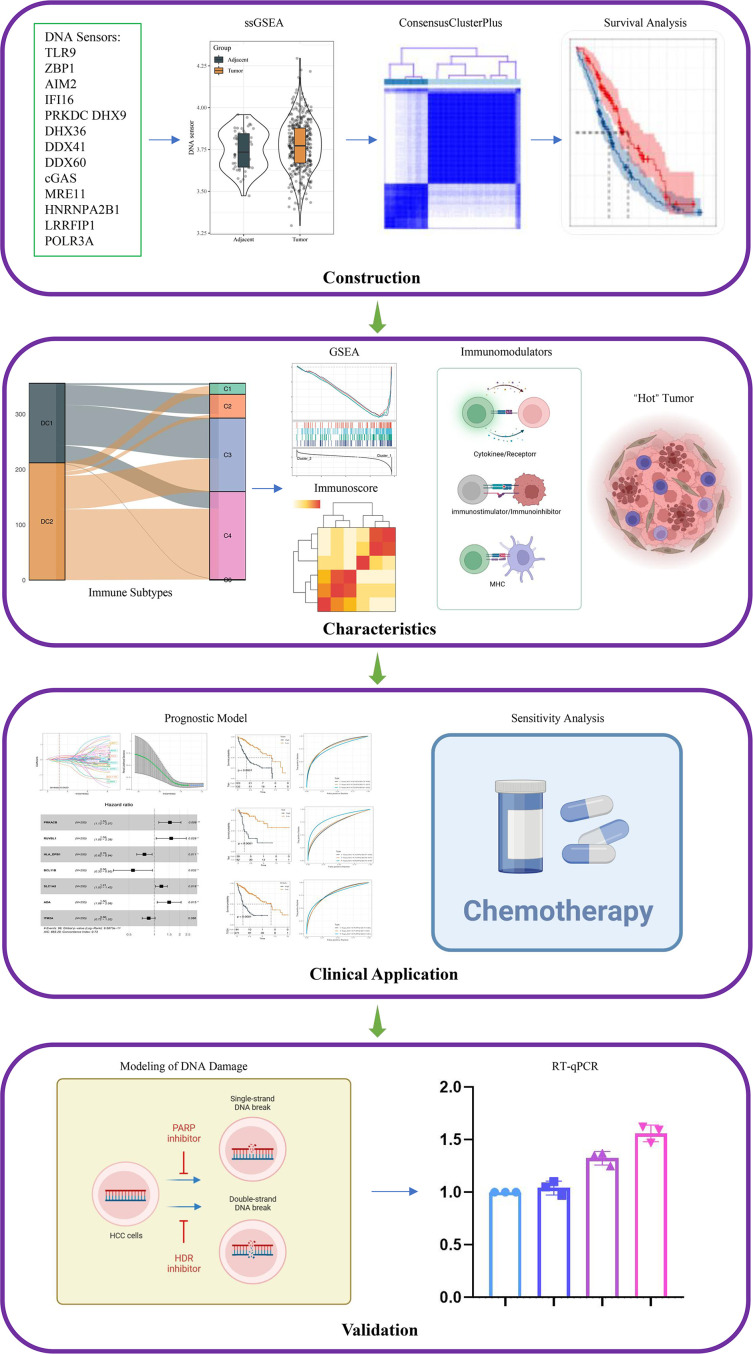
Flowchart of this study.

In the present study, we selected 14 genes, including *TLR9, ZBP1, AIM2, IFI16, PRKDC, DHX9, DHX36, DDX41, DDX60, cGAS, MRE11, HNRNPA2B1, LRRFIP1* and *POLR3A*, as DNA sensors [13–16], and downloaded corresponding gene expression files and clinical data of HCC patients from TCGA-LIHC, HCCDB18 and GSE76427 datasets. The relationship between the DNA sensors and the prognosis of HCC patients was explored. In addition, the DNA sensors were employed to classify HCC into multiple molecular subtypes, and the immunophenotypic characteristics across the subtypes were comparatively analyzed. Moreover, a prognostic model was established based on the DNA sensors and related genes, and the association between the DNA sensors and chemotherapeutic drug sensitivity was investigated. This study hopes to bring new insights into the immunotherapy for HCC. The workflow for this study is shown in Figure 1.

## RESULTS

### Molecular subtypes identification and prognostic analysis

**Figure 2:**
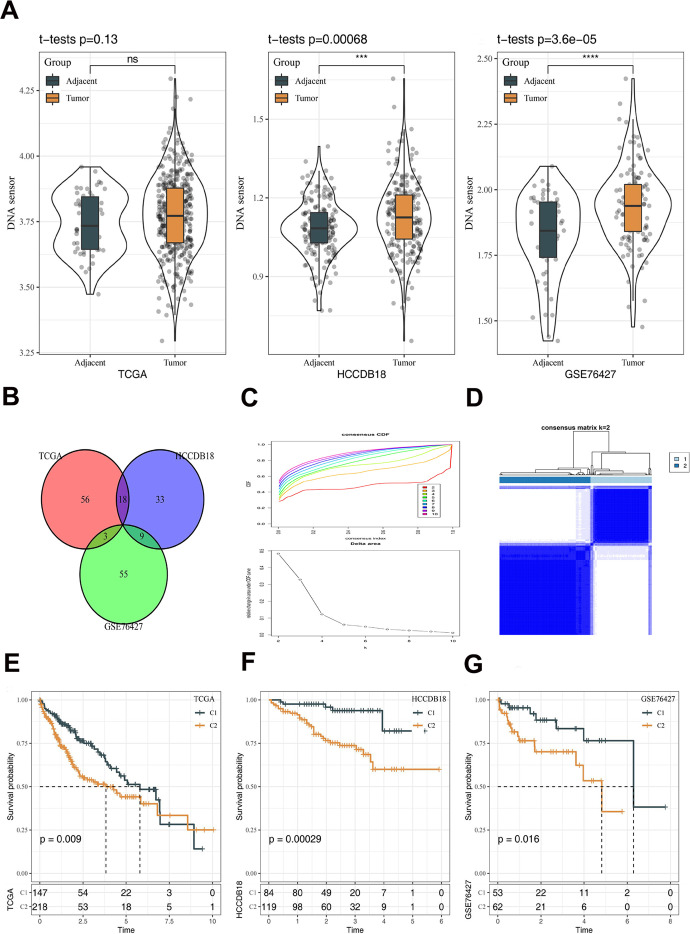
**Construction of molecular subtypes and prognostic analysis.** (**A**) The differences of DNA sensors expression between tumor and adjacent tissues. (**B**) Venn diagram revealing 30 genes in relation to prognosis of HCC by univariate COX analysis. (**C**) CDF curves and CDF delta area curves. The optimal number of clusters is the K value where the CDF curve is the smoothest and also the turning point of the Delta area. (**D**) Subtype classification of HCC samples. (**E**) Prognostic analysis in TCGA dataset. (**F**) Prognostic analysis in HCCDB18 dataset. (**G**) Prognostic analysis in GSE76427 dataset. The line in the box represents the median value, and the asterisks represent the P-value (***p < 0.001; ****p < 0.0001). The statistical analyses were performed by the student t-test. HCC, hepatocellular carcinoma; CDF, cumulative distribution function; ns, not significant.

Expression of the selected DNA sensors in HCC and normal tissue samples derived from the TCGA-LIHC, HCCDB18 and GSE76427 datasets were comparatively analyzed with ssGSEA. Higher scores were found in HCC tissue samples, and those in the HCCDB18 and GSE76427 datasets were statistically different between the two types of samples (Figure 2A). Differential analysis of mRNA also found that DNA sensor genes were highly expressed in tumor tissues compared to adjacent tissues (Supplementary Figure 1). The analysis of DNA methylation results reveals a correlation between the DNA sensor genes' methylation status and various factors such as race, age, and BMI in patients with liver cancer. Furthermore, these findings demonstrate that the methylation status of DNA sensor genes can impact the prognosis of patients (Supplementary Figures 2, 3). In addition, correlation analysis between the DNA sensors and protein-coding genes was performed, resulting in 319 DNA sensors-related genes. Further analysis revealed that DNA sensors-related genes involved in biological functions included regulation of lymphocyte activation, leukocyte migration and T cell activation, etc. Their underlying molecular mechanisms are related to cytokines, including cytokine receptor activity, cytokine receptor binding and cytokine−cytokine receptor interaction (Supplementary Figure 4). Among the DNA sensors-related genes, 30 genes in relation to prognosis of HCC in at least two of the three datasets were screened out by univariate COX analysis (Figure 2B). Consensus clustering was conducted in HCC samples from the TCGA-LIHC dataset, and k = 2 was selected based on the CDF, resulting in 2 stable clusters: C1 and C2 (Figure 2C, 2D). Survival analysis in the TCGA-LIHC, HCCDB18 and GSE76427 datasets demonstrated that the survival between the C1 and C2 subtypes of HCC was distinctly different (Figure 2E–2G).

### Clinical and immune characteristics of two molecular subtypes

**Figure 3:**
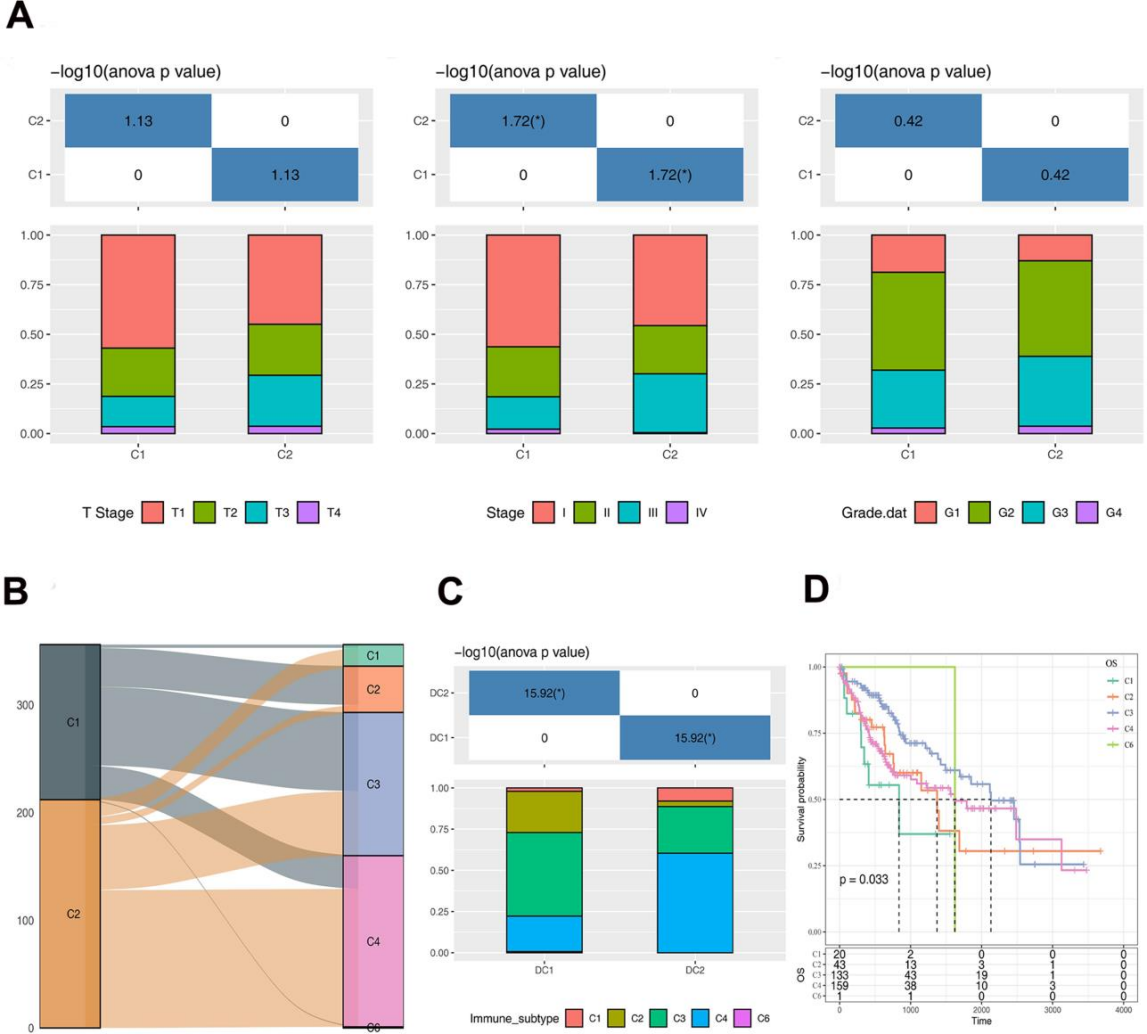
**Analysis of clinical and immunological characteristics of C1 and C2 subtypes.** (**A**) The distribution of clinical characteristics of C1 and C2 subtypes. (**B**) Sankey Diagram showing the distribution of immune subtypes in C1 and C2. (**C**) The differences of immune subtypes distribution between C1 and C2. (**D**) Survival curve analysis of immune subtypes. The asterisks represent the P-value (*p < 0.05). The statistical analyses were performed by the one-way ANOVA.

The clinical features of the two subtypes (C1 and C2) were comparatively analyzed in the TCGA-LIHC dataset. Significant difference was found in Stage between the C1 and C2 subtypes, whereas the T stage and Grade marginally varied (Figure 3A). There are 6 immune subtypes according to the literature [17], including C1: wound healing, C2: IFN-γ dominant, C3: inflammatory, C4: lymphocyte depleted, C5: immunologically quiet, and C6: TGF-β dominant, among which the C1, C2 and C6 immune subtypes are associated with poor prognosis. As stratified by the 6 immune subtypes, we found that the subtype C1 was dominantly by C3 immune subtype, while the subtype C2 was dominantly by C4 immune subtype (Figure 3B, 3C). Moreover, survival difference was observed between different immune subtypes (Figure 3D).

### GSEA and immune scoring

**Figure 4:**
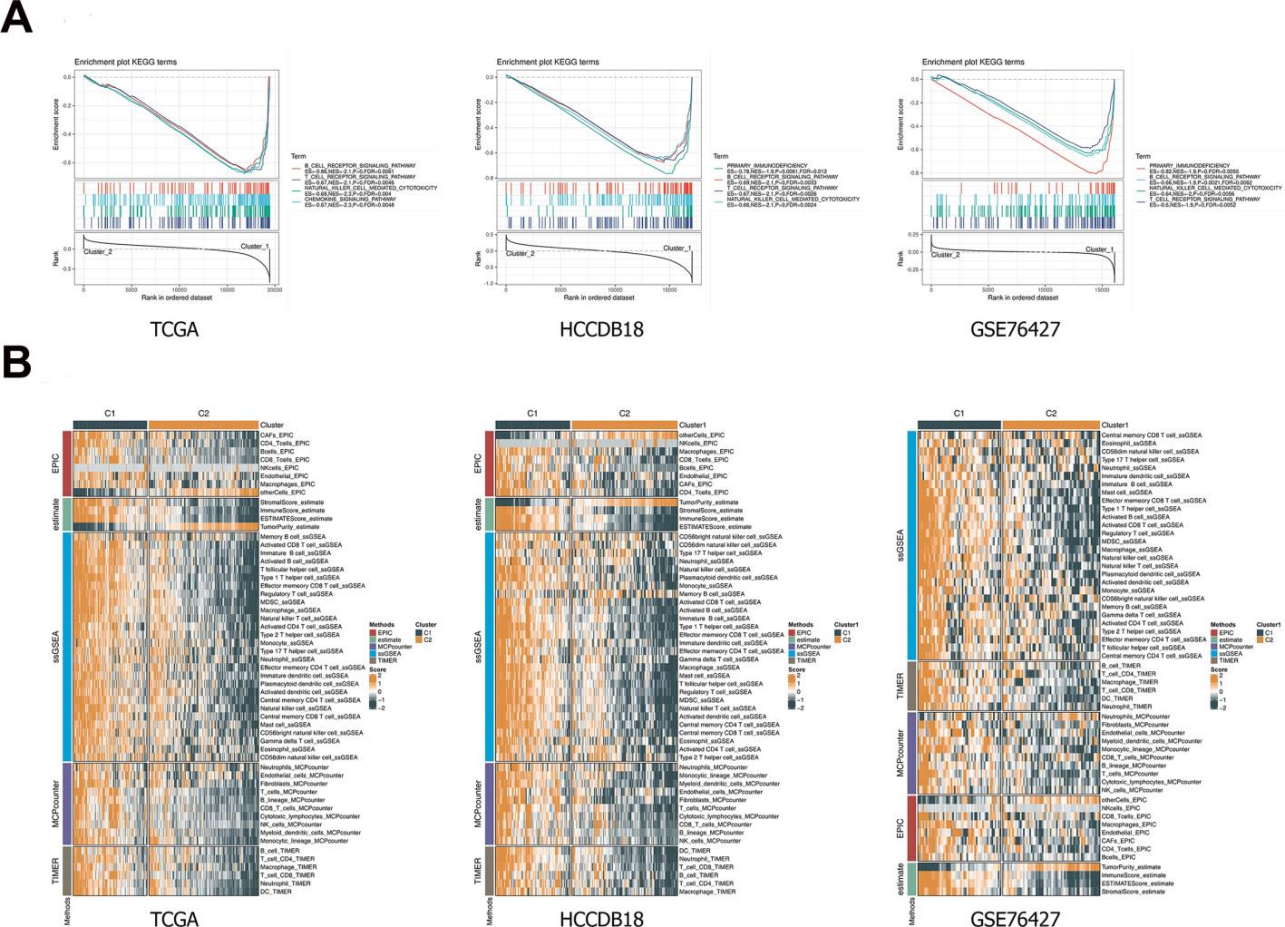
**GSEA and immune scoring.** (**A**) The pathway analysis of the C1 and C2 subtypes was performed using GSEA. (**B**) Comparison of immune scores between C1 and C2 subtypes. Heatmap illustrated immune scores among two classifications. Orange represents high scores, and green represents low scores. GESA, gene set enrichment analysis.

The pathway analysis of the C1 and C2 subtypes was performed using GSEA in the TCGA-LIHC, HCCDB18 and GSE76427 datasets. Pathways, including chemokine signaling pathway, B cell receptor signaling pathway, T cell receptor signaling pathway, and natural killer cell mediated cytotoxicity, were significantly enriched in C1 subtype (Figure 4A). In addition, all EPIC, ESTIMATE, ssGSEA, MCPcounter and TIMER results demonstrated higher immune scores of the C1 subtype than the C2 subtype, and the differences were statistically significant (Figure 4B).

### Immunomodulatory function of C1 and C2 subtypes

**Figure 5:**
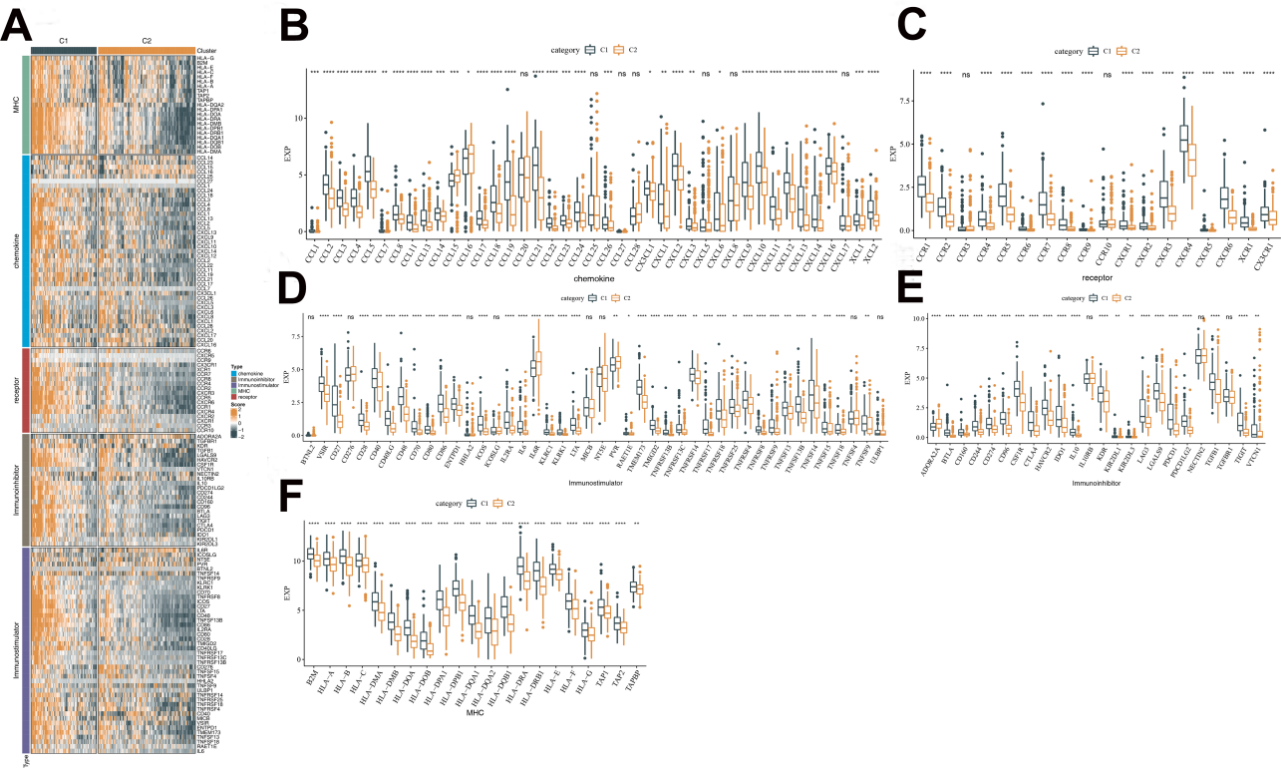
**Analysis of the immunomodulatory functions of C1 and C2 subtypes.** (**A**) Differences in immunomodulator between subtypes C1 and C2 shown in a heatmap and orange represents high scores, and green represents low scores. (**B**) Differential expression of chemokines between C1 and C2 in TCGA cohort. (**C**) Differential expression of chemokine receptor between C1 and C2 in TCGA cohort. (**D**) Differential expression of immunostimulatory between C1 and C2 in TCGA cohort. (**E**) Differential expression of immunoinhibitor between C1 and C2 in TCGA cohort. (**F**) Differential expression of MHC between C1 and C2 in TCGA cohort. The line in the box represents the median value, and the asterisks represent the P-value (*p < 0.05; **p < 0.01; ***p < 0.001; ****p < 0.0001). The statistical analyses were performed by the student t-test. TCGA, the cancer genome atlas; MHC, major histocompatibility complex.

Five groups of genes with immunomodulatory functions, including chemokine, chemokine receptor, immunostimulator, immunoinhibitor and MHC, were confirmed from the literature [18]. Expression analysis in the TCGA-LIHC dataset found that most of the genes exhibited differential expression between the C1 and C2 subtypes, and that these genes tended to express more abundantly in C1 subtype (Figure 5). Consistent results were found in the HCCDB18 and GSE76427 datasets (Supplementary Figure 5).

### “Hot” tumor microenvironmental characteristics of C1 and C2 subtypes

**Figure 6:**
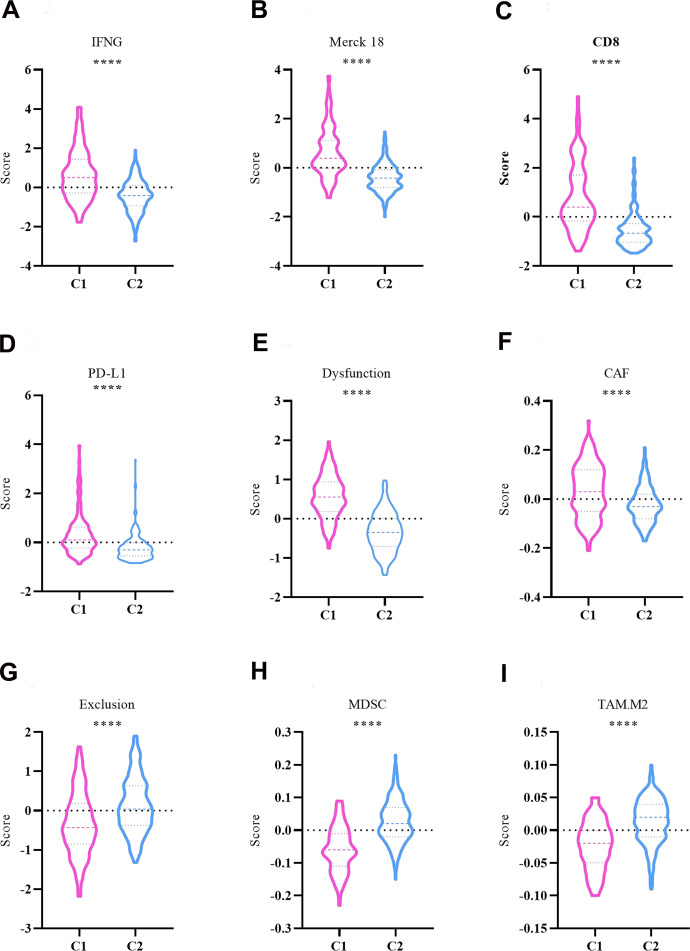
**Comparison of ‘hot’ tumor characteristics between C1 and C2 subtypes.** (**A**) IFNG. (**B**) Merck 18. (**C**) CD8. (**D**) PD-L1. (**E**) Dysfunction. (**F**) CAF. (**G**) Exclusion. (**H**) MDSC. (**I**) TAM.M2. The asterisks represent the P-value (****p < 0.0001). The statistical analyses were performed by the student t-test. IFNG, interferon-γ; CD8, cluster of differentiation 8; PD-L1, programmed cell death 1 ligand 1; CAF, cancer associated fibroblast; MDSC, myeloid-derived suppressor cells; TAM, tumor associated macrophage.

The “Hot” tumor feature of C1 and C2 subtypes was analyzed with IFN-γ, Merck 18, CD8, PD-L1, T-cell dysfunction score, T-cell exclusion score, MDSC score, CAF score, and TAM.M2 score using the TIDE tool in the TCGA-LIHC datasets. In comparison to the C2 subtype, the C1 subtype had significantly higher levels of IFN-γ, Merck 18, CD8, PD-L1, T-cell dysfunction score, and CAF score (Figure 6A–6F). In contrast, distinctly higher levels of T-cell exclusion score, MDSC score and TAM.M2 score were found in the C2 subtype (Figure 6G–6I).

### Establishment of DNA sensors-related prognostic model

**Figure 7:**
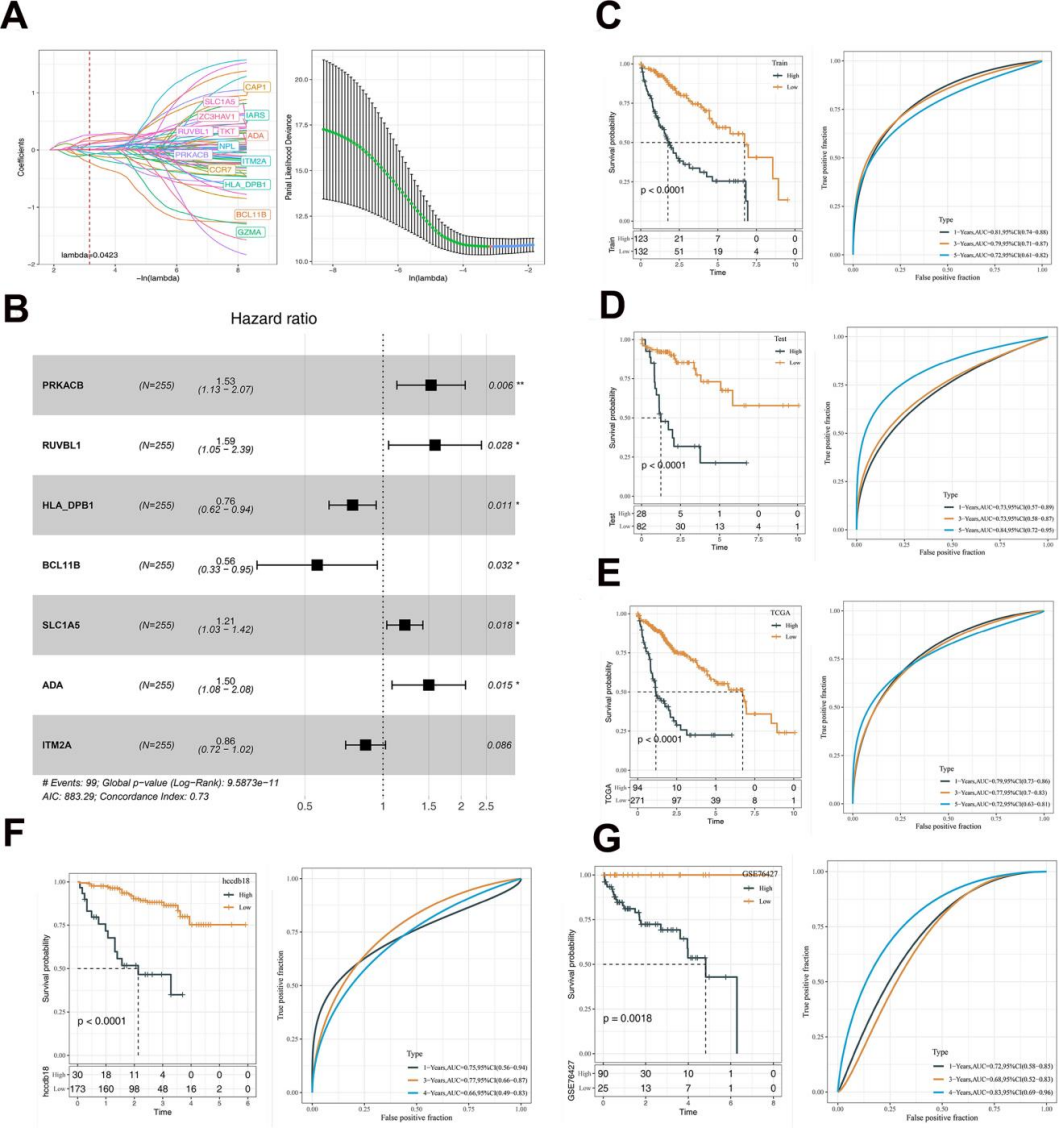
**The prognostic model was constructed based on DNA sensors related genes.** (**A**) LASSO coefficient profiles of 14 prognostic mRNAs in TCGA training cohort and the coefficient profile plot was developed against the log (Lambda) sequence. When lambda is 0.0423, the smallest value, fourteen genes are obtained. (**B**) Multi-variate analyses. (**C**) Analyses of KM and ROC on TCGA training data. (**D**) The KM and ROC analysis of the model on the TCGA validation dataset. (**E**) The KM and ROC analysis of the model on the TCGA dataset. (**F**) The KM and ROC analysis of the model on the HCCDB18 dataset. (**G**) The KM and ROC analysis of the model on the GSE76427 dataset. The asterisks represent the P-value (*p < 0.05, **p < 0.01). The statistical analyses were performed by the Kaplan-Meier analysis. LASSO, Least absolute shrinkage and selection operator; TCGA, the cancer genome atlas; KM, Kaplan–Meier; ROC, receiver operating characteristic.

TCGA samples were assigned into the training or test set with the method of random sampling (7:3). Univariate analysis in the training set resulted in 69 prognosis-related genes (p < 0.05), while LASSO model was used to obtain 14 genes with the lambda that yields the minimum mean squared error of 0.0423 (Figure 7A). Univariate Cox regression analysis showed that 7 DNA sensors-related genes were associated with the survival of HCC patients (Figure 7B). A corresponding risk score was generated and divided samples into the high- and low-risk groups. Kaplan-Meier survival and ROC curves in the TCGA training set (Figure 7C), TCGA test set (Figure 7D), TCGA-LIHC total set (Figure 7E), HCCDB18 dataset (Figure 7F), and GSE76427 (Figure 7G) dataset were generated. It was noted that the overall survival (OS) in the high-risk group was remarkably lower than that in the low-risk group. Additionally, the 7-gene signature was capable of effectively predicting the OS of HCC patients at 1-, 3-, and 5-years.

### Chemotherapeutic drug sensitivity analysis and small molecular compounds prediction

**Figure 8:**
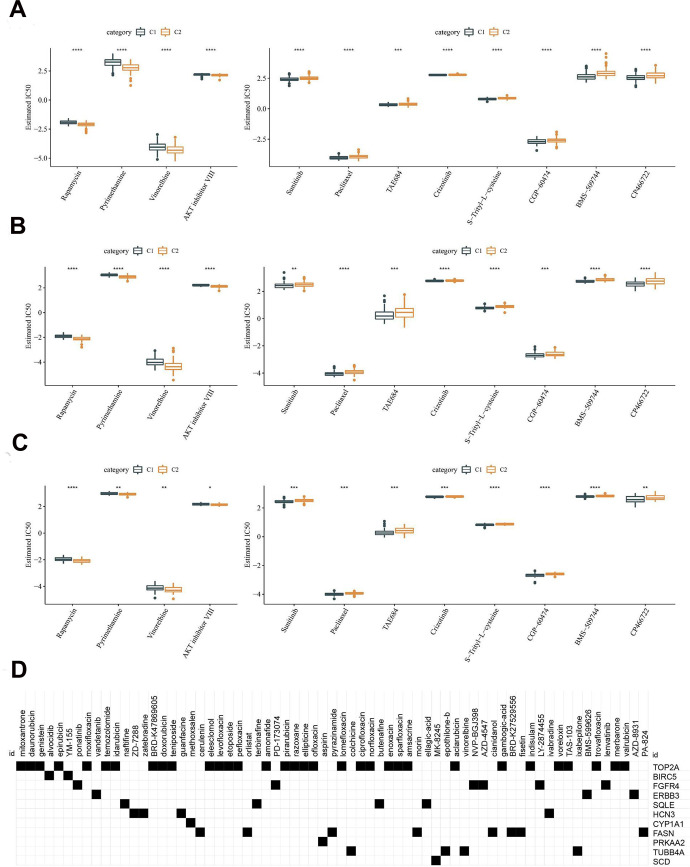
**Difference analysis of chemotherapeutic drug sensitivity.** (**A**) Analysis of chemosensitivity in TCGA dataset. (**B**) Analysis of chemosensitivity in HCCDB18 dataset. (**C**) Analysis of chemosensitivity in GSE76427 dataset. (**D**) CMap database analysis identified candidate drugs targeting the two molecular subtypes based on the DEGs. The line in the box represents the median value, and the asterisks represent the P-value (*p < 0.05, **p < 0.01, ***p < 0.001, ****p < 0.0001). The statistical analyses were performed by the student t-test.

Linkage between the molecular subtypes of HCC and the sensitivity to chemotherapeutic agents was analyzed in the TCGA-LIHC, HCCDB18 and GSE76427 datasets. Consistent in the three datasets, the C1 subtype was more sensitive to Sunitinib, Paclitaxel, TAE684, Crizotinib, S-Trityl-L-cysteine, CGP-60474, BMS-509744 and CP466722, whereas the C2 subtype was more sensitive to Rapamycin, Pyrimethamine, Vinorelbine and AKT inhibitor VIII (Figure 8A–8C). Using the TCGA dataset, differentially expressed analysis was first performed between the HCC subtypes C1 and C2. A total of 890 DEGs were identified, consisting of 57 upregulated genes and 833 downregulated genes (Supplementary Table 1). Subsequently, these genes were uploaded to the CMap database, and compounds with an absolute enrichment value greater than 0.5 were retained. Ultimately, 78 small molecular compounds with potential therapeutic value were identified (Figure 8D).

### The expression of DNA sensors was verified by RT-qPCR

**Figure 9:**
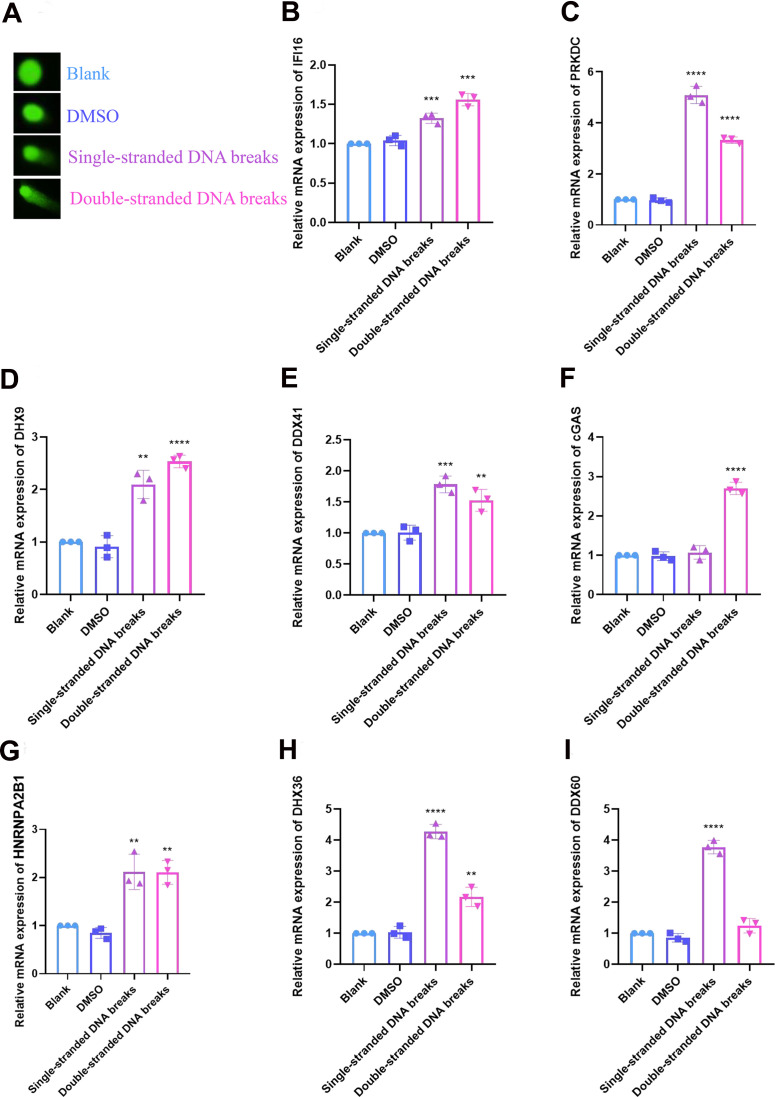
**Validation of DNA sensors in cellular DNA damage models was performed by RT-qPCR.** (**A**) DNA damage was verified by comet assay. (**B**) Relative mRNA expression of IFI16. (**C**) PRKDC. (**D**) DHX9. (**E**) DDX41. (**F**) cGAS. (**G**) HNRNPA2B1. (**H**) DHX36. (**I**) DDX60. The experimental data were represented by mean ± SD from at least three independent experiments, and the asterisks represent the P-value (**p < 0.01, ***p < 0.001, **** p < 0.0001). The statistical analyses were performed by the student t-test. RT-qPCR, real-time quantitative polymerase chain reaction; IFI16, interferon gamma inducible protein 16; PRKDC, protein kinase, DNA-activated, catalytic subunit; DHX9, DExH-box helicase 9; DDX41, dead-box helicase 41; cGAS, cyclic GMP-AMP synthase; HNRNPA2B1, heterogeneous nuclear ribonucleoprotein A2/B1; DHX36, deah-box helicase 36; DDX60, DExD/H-Box helicase 60.

A model of DNA damage in HCC cells was established using Olaparib or YU238259, an inhibitor for homology-direct repair (HDR), and the degree of DNA damage was determined via the comet assay. The mRNA expression of 8 DNA sensors, including *IFI16, PRKDC, DHX9, DDX41, cGAS, HNRNPA2B1, DHX36* and *DDX60*, was examined by RT-qPCR. The result of comet assay indicated that both Olaparib and YU238259 induced DNA damage in HCC cells after 24 hours of treatment (Figure 9A). In cells treated by Olaparib, the DNA sensors, except for *cGAS*, exhibited significantly increased expression levels. While in cells treated with YU238259, except for DDX60, the DNA sensors had distinctly up-regulated expression (Figure 9B–9I).

## DISCUSSION

HCC is a global threat to human health, and its incidence has continued to increase [19]. Unfortunately, biomarkers that can help for clinical decision-making and guide HCC treatment are limited [20]. By now, serum alpha-fetoprotein (AFP) is the only one recognized biomarker available to predict the poor outcomes of HCC in all stages, and its level is linked with the VEGF pathway activation in HCC [21]. To improve the treatment and prognosis of HCC, it is significant to clarify the molecular pathogenesis of HCC and identify different molecular subtypes that result from its heterogeneity. DNA damage is critical in progression from chronic liver disease to HCC, which indicates the potential value of the genes and pathways related to DNA damage response/repair in HCC treatment and prognosis [22]. Research revealed that DNA damage repair-related genes were capable of dividing HCC into two subtypes with varying genomic features and prognoses [23]. Another study reported a risk scoring system for HCC based on homologous recombination deficiency (HRD)-related genes, which could be employed to predict the prognosis of HCC patients [24]. The activation of the cGAS/STING pathway by cytoDNA recognition has been reported to benefit prognosis and immunotherapy response in patients with HCC [25, 26]. This benefit results from the release of inflammatory factors, such as type I interferon, following activation of the cGAS-STING pathway [27]. However, it remains unclear whether other DNA sensors can regulate inflammatory cytokine release and serve as prognostic markers in HCC. In the present study, we focused on DNA sensors for analysis. Based on the expression of DNA sensors-related genes, two molecular subtypes of HCC were identified: C1 and C2, and the OS of patients with type C1 HCC was better than that of patients with type C2 HCC. We believe that DNA sensors are potentially valuable biomarkers that can be employed to predict the survival outcomes of HCC patients and its underlying mechanisms are related to the regulation of cytokine receptor activity, cytokine receptor binding and cytokine−cytokine receptor interaction.

For the past few years, techniques of conjoint analysis based on genomics [28], proteomics [29] and multi-omics [30] have made progress in research on HCC molecular typing. However, clinical translation remains currently missing. ICIs targeting immune checkpoints PD-1/CTLA-4 have been proved for treatment of HCC [31]. In this context, immunotyping in different molecular subtypes of HCC may be a novel approach instructive to clinical treatment [32]. Here, the C1 and C2 molecular subtypes of HCC were further immunotyped, revealing a predominance of C3 immune subtype (inflammatory) in the C1 molecular subtype while there is a predominance of C4 immune subtype (lymphocyte depleted) in the C2 molecular subtype. According to research, HCC can be divided in two major immune classes, Inflamed and non-inflamed. An Inflamed class is characterized by high levels of chemokine and of CD8+ T cell [33]. Recently, the Inflamed class was demonstrated to be enriched in patients with HCC who responded to anti-PD-1/PD-L1 antibodies [34]. Enrichment analysis of this study also demonstrated that immune pathways, such as Chemokine Signaling Pathway, B Cell Receptor Signaling Pathway, T Cell Receptor Signaling Pathway and Natural Killer Cell Mediated Cytotoxicity were significantly enriched in the C1 subtype. Additionally, five immune scoring methods consistently revealed higher scores in the C1 subtype than the C2 subtype. Presently, the response rate of HCC to immunotherapy remains low in spite of certain clinical benefits [35], while the immune microenvironment in HCC represents as one of the most important factors influencing the response rate [36]. According to the present study, the C1 subtype is characterized by inflammation with high levels of chemokine signaling and might benefit from immunotherapy.

The combination of ICIs and other therapies has been applied in clinical tumor treatment with satisfactory results, whereas the response rate is still limited [37]. Consistently, limited response rate is also seen in the combination therapy with Atezolizumab and Bevacizumab, a systemic treatment for HCC currently [38]. In this context, it is imperative to look for new molecules that can be used in combination immunotherapies for HCC. Referring to the previous literature, chemokine/chemokine receptor [39], immunostimulator/immunoinhibitory [40] and MHC molecules [41, 42] are important participants in the interplay between tumor cells and the immune system. Such interplay usually leads to dynamic immunoediting process, enhancing immune response or inducing immune escape [43]. Therefore, we analyzed the expression of these molecules in the C1 and C2 subtypes. It was found that most of the chemokines/receptors were expressed higher in the C1 subtype than the C2. Further evidence for C1 subtype’s proinflammatory properties was provided by this result. Moreover, immunostimulator, immunoinhibitor and MHC molecules also had higher levels of C1 than C2. The results indicated that HCC patients with C1 subtype might have different immune response in immunotherapy with C2 subtype.

According to the response to immunotherapy, solid tumors can be classified into two categories: “Hot (immune-inflamed)” and “Cold (immune-desert/immune-excluded)” [44]. For “Hot” tumors, the major feature falls into T-lymphocyte infiltration [45]. A growing number of studies have suggested that tumor patients with T-lymphocyte infiltration may also poorly respond to ICIs therapy [46]. Therefore, T-lymphocyte infiltration is not sufficient for identifying the group of patients who may be highly responsive to ICIs. This warrants us to know more about the microenvironment features of “Hot” tumors, as this can help us clarify the mechanism underlying the response to ICIs immunotherapy [47]. Thus, the present study also explored the “Hot” tumor microenvironment features in different molecular subtypes with IFN-γ, Merck 18, CD8, PD-L1, T-cell dysfunction score, T-cell exclusion score, MDSC score, CAF score, and TAM.M2 score. In comparison to the C2 subtype, the C1 subtype had significantly higher levels of IFN-γ, Merck 18, CD8, PD-L1, T-cell dysfunction score, and CAF score. As IFN-γ, Merck 18, CD8 and PD-L1 are linked with tumor immune escape, the C1 subtype might be at a higher risk of developing immune escape [48]. This is also evidenced by the higher T-cell dysfunction score and CAF score in the C1 subtype [49, 50]. More crucially, the finding suggested that the C1 subtype may benefit more from anti-PD-L1 therapy than the C2 subtype, because of the effect of anti-PD-L1 antibodies that blocks the inhibitory effect of PD-L1 on T cells and thereby recovers the cellular biological functions [51]. While in the C2 subtype, T-cell exclusion score, MDSC score and TAM.M2 score were much higher, which implied a low T-cell density in C2 tumors while there are more immunosuppressive cells (including MDSC and TAM.M2). This also indicated that patients with C2 HCC were less likely to gain benefit from ICIs immunotherapy. Combining the results, the C1 subtype of HCC presented with microenvironment features of “Hot” tumors. DNA sensors might be a new class of biomarkers that can be employed to predict the response to ICIs immunotherapy.

Besides, our study constructed a prognostic model based on 7 DNA sensors-related genes, including *PRKACB, RUVBL1, HLA-DPB1, BCL11B, SCL1A5, ADA, ITM2A*, to analyze the clinical value of DNA sensors. The model performed well in predicting the 1-, 3- and 5-year OS in HCC patients.

The current study still has the following limitations that need to be addressed. First, this study only examined the expression of DNA sensors upon DNA damage using RT-qPCR, whereas the underlying molecular mechanism requires further experimental validation. Second, the expression of DNA sensors and their prognostic significance in HCC patients have not been validated in clinical settings.

Taken together, this study identified two molecular subtypes of HCC (C1 and C2) based on the DNA sensors, which varied significantly in terms of the immune features, including the inflamed characteristics, immune microenvironment scores, and “Hot” tumor microenvironment property. The findings of the study provide evidence for the potential of the combination of DNA sensors targeting therapy with ICIs immunotherapy as a therapeutic strategy for HCC. In addition, the DNA sensors were also proven with favorable performance in predicting the survival outcomes of HCC patients. In all, this study provides new insights into the markers available for predicting prognosis and immunotherapy response for HCC.

## MATERIALS AND METHODS

### Data source and preprocessing

RNA-seq data (FPKM format) were downloaded from the TCGA-LIHC dataset with the UCSC Xena browser (https://xenabrowser.net/) and then converted to TPM format. GSE76427 dataset was derived from the GEO database (https://www.ncbi.nlm.nih.gov/geo/). HCCDB18 dataset was obtained from the HCCDB website (http://lifeome.net/database/hccdb/download.html). All the three datasets contained clinical data of HCC patients.

### Single-sample gene set enrichment analysis (ssGSEA) and functional analysis

ssGSEA calculates an enrichment score as the weighted sum of the difference between the empirical Cumulative Distribution Function (CDF) of the genes within a gene set and the remaining genes in a single sample, and then provides a normalized value based on the difference between the minimal and maximal expression values. Here, the enrichment scores of the DNA sensors in the TCGA-LIHC, GSE76427 and HCCDB18 datasets were calculated. Pearson correlation was conducted to obtain protein-coding genes associated with the DNA sensors in the TCGA-LIHC, GSE76427 and HCCDB18 datasets, with the threshold set as |Pearson’s R| > 0.5 and p < 0.05. Gene Ontology (GO) and Kyoto Encyclopedia of Genes and Genomes (KEGG) analyses were conducted to explore potential molecular processes and biological pathways related to DNA sensors in HCC. These analyses were performed using the R package “WebGestaltR”. The p < 0.05 were considered statistically significant.

### Analysis of DNA sensor genes methylation and prognosis

MethSurv online tool was used (version MethSurv©2017, https://biit.cs.ut.ee/methsurv/). It is a web tool to provide survival analysis based on DNA methylation biomarkers using TCGA data. The methylation and prognostic value of DNA sensor genes in LIHC were analyzed by MethSurv online tool.

### Identification of DNA sensors-related molecular subtypes of HCC

The DNA sensors-related genes were subjected to univariate COX analysis in the TCGA-LIHC, GSE76427 and HCCDB18 datasets. Candidate genes were selected as the genes associated with prognosis of HCC in at least two datasets (p < 0.05). Consensus clustering analysis was then performed based on the candidates using the package “ConsensusClusterPlus”, and the optimal number of clusters was determined via CDF. The Kaplan–Meier (K–M) survival curves of overall survival (OS) were used to evaluate the clinical prognostic value of DNA sensors-related genes.

Clinical features of different molecular subtypes were comparatively analyzed from T stage, Stage and Grade. There are 6 immune subtypes according to the literature, including C1: wound healing, C2: IFN-γ dominant, C3: inflammatory, C4: lymphocyte depleted, C5: immunologically quiet, and C6: TGF-β dominant [17]. The difference in the distribution of the 6 immune subtypes in different molecular subtypes was observed.

### GSEA and immune scoring

The KEGG gene set c2.cp.kegg.v7.0.symbols.gmt containing 186 signaling pathways was selected, and the significant enriched pathways in different molecular subtypes were revealed by GSEA with the threshold set as p < 0.05 and FDR < 0.25. Immune scores in different molecular subtypes were calculated by EPIC, MCPcounter, TIMER, ESTIMATE and ssGSEA, and the results were shown as a Heatmap. Five groups of genes with immunomodulatory functions, including chemokine, chemokine receptor, immunostimulator, immunoinhibitor, and MHC, were obtained from the literature [18]. Expression of these genes in different molecular subtypes was analyzed.

### “Hot tumor” feature in different molecular subtypes of HCC

The “Hot tumor” features in different molecular subtypes were comparatively analyzed according to the levels of T-cell dysfunction score, T-cell exclusion score, IFN-γ, Merck18, CD8, MDSC, CAF and TAM.M2 using the TIDE tool (http://tide.dfci.harvard.edu/).

### Construction and validation of prognostic risk signature

TCGA samples were assigned into the training and test sets at 7:3 using the random sampling method. The DNA sensors-related genes were subjected to univariate COX analysis in the training set, yielding prognosis-related genes (p < 0.05). Candidate signature genes were obtained via LASSO regression and multivariate COX analysis in succession, and the corresponding risk coefficients were acquired. Each sample in the TCGA-LIHC training and test sets, GSE76427 dataset and HCCDB18 dataset were assigned a risk score and divided into the high- or low-risk group. Kaplan-Meier and ROC curves were generated to analyze the survival difference between the two groups.

### Analysis of sensitivity to chemotherapeutic agents

R package “pRRophetic” was used to predict the half maximal inhibitory concentration (IC50) of chemotherapeutic agents in different molecular subtypes in the TCGA-LIHC, HCCDB18 and GSE76427 datasets.

### Small molecular compounds prediction for the treatment of hepatocellular carcinoma

To predict the small molecule compounds of HCC, the differentially expressed genes (DEGs) between subtypes C1 and C2 were initially identified using the limma R package with | logFC| > 1.5 and adjusted p < 0.05. Subsequently, these DEGs were uploaded to the Connective Map (CMap) database (https://clue.io/). The small molecule compounds were predicted based on the enrichment value and p-value.

### Cell culture and quantitative real-time PCR (RT-qPCR)

Human HCC cell line HUH7 was provided by the Stem Cell Bank of Chinese Academy of Sciences. Cell culture was performed in DMEM (Gibco, USA) medium containing 10% fetal bovine serum at 37° C with 5% CO2. The cells were intervened by Olaparib or YU238259 (inhibitor for HDR) for 24 h and were then harvested for DNA extraction. DNA damage analysis was conducted according to the procedure described by Comet Assay Kit (iPhase Pharma Services, China). All samples were stained with SYBR Green I for scoring. The percentage of DNA in tail was scored using CASP software in 90 random nuclei per sample. All experiments were carried out in three replicates.

Total RNA extraction was completed using the RNeasy Mini Kit (Magen, China), and cDNA synthesis was fulfilled with the PrimeScript™ RT Master Mix (Takara, China). Primers were designed and synthesized by Takara. RT-qPCR was ran using the TB Green® Premix Ex Taq™ II (Takara, China) with the LightCycler 480 System (Roche, Switzerland). The results were shown as 2-ΔΔCt. The mRNA expression of 8 DNA sensors, including *IFI16, PRKDC, DHX9, DDX41, cGAS, HNRNPA2B1, DHX36* and *DDX60*, were examined, and *β-actin* was selected as the internal reference gene. All experiments were carried out in three replicates. Sequences of primers were detailed in Supplementary Table 2.

### Statistical analysis

All statistical analysis was conducted using R software (version 4.03) and SPSS20.0 (IBM Corp, USA). The experimental data were represented by mean ± standard deviation (SD) from at least three independent experiments. Comparison between two groups was performed using Student t-test, whereas comparison among over two groups was conducted with one-way ANOVA. Kaplan-Meier together with log-rank test was used to generate survival curves. Two-sided p < 0.05 was considered statistically significant.

## Supplementary Material

Supplementary Figures

Supplementary Table 1

Supplementary Table 2
