# Supplementary material for: Molecular subtypes based on DNA sensors predict prognosis and tumor immunophenotype in hepatocellular carcinoma
Source: Aging (Albany NY). 2023 Jul 14;15(14):6798–821. doi: 10.18632/aging.204870 (PMC10415551; doi:10.18632/aging.204870)
Supplement: Supplementary Figures [file aging-15-204870-s001.pdf]

SUPPLEMENTARY FIGURES

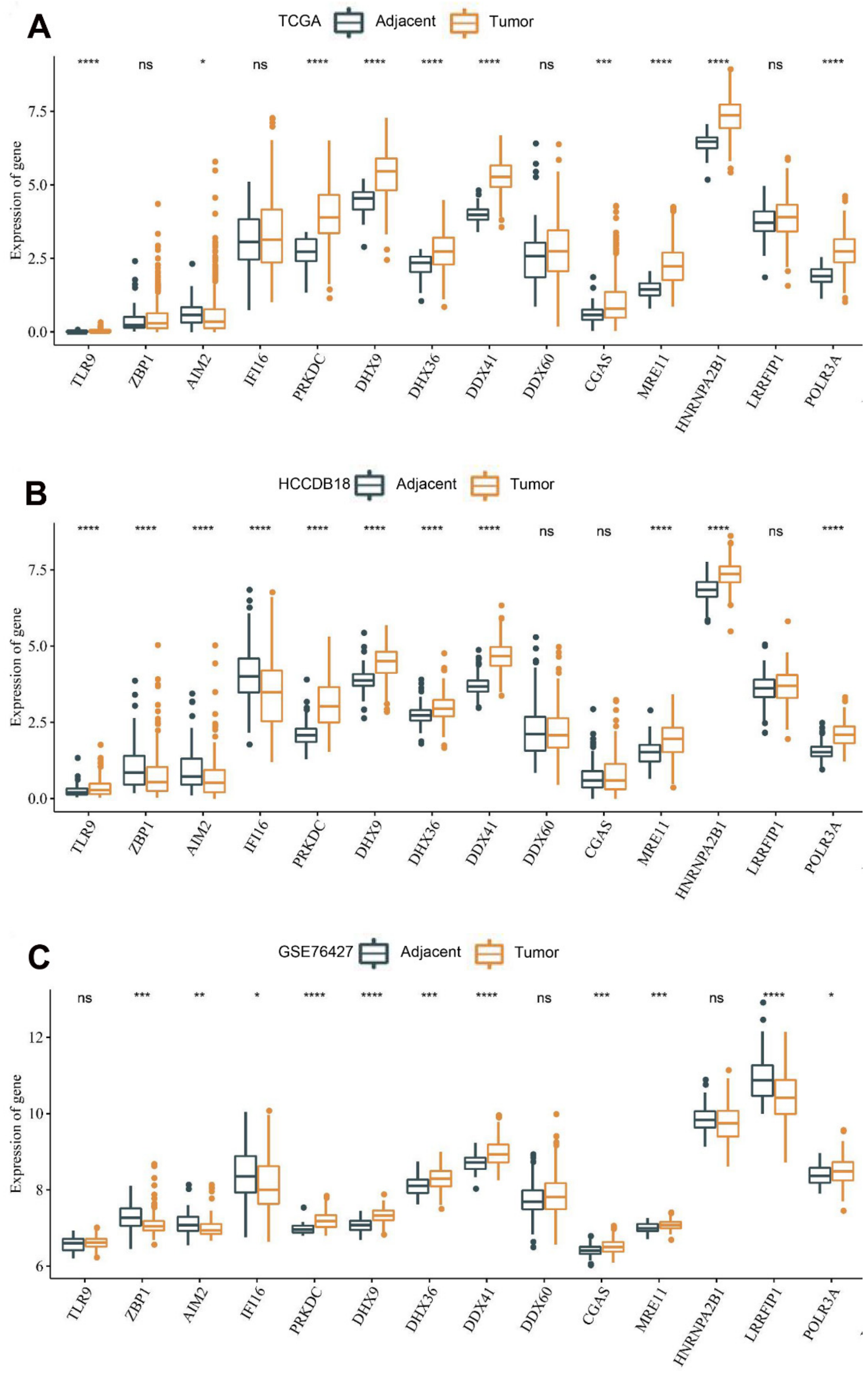

Supplementary Figure 1. Analysis of mRNA differences in DNA sensor genes. (**A**) TCGA dataset. (**B**) HCCDB18 dataset. (**C**) GSE76427 dataset.

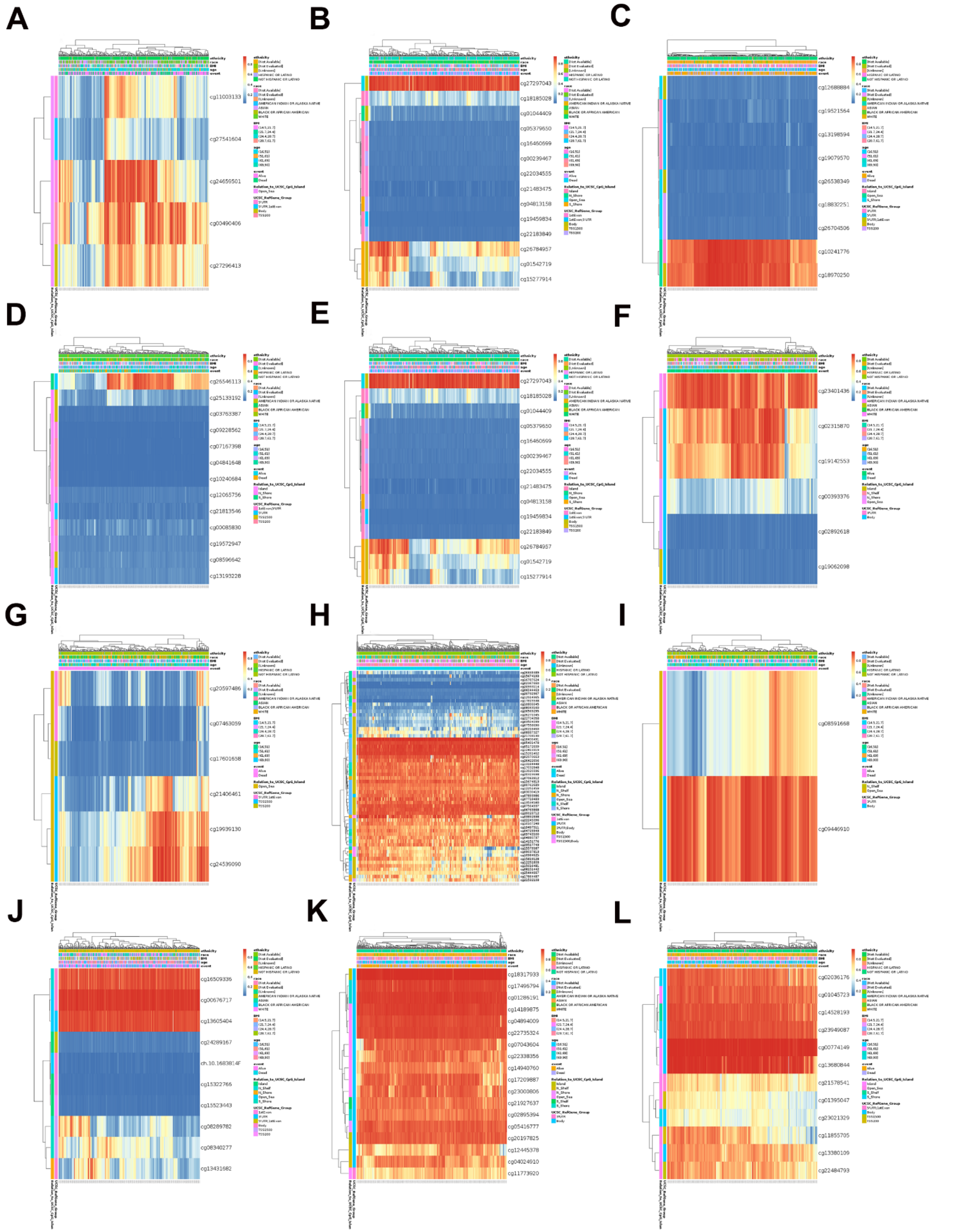

Supplementary Figure 2. Analysis of DNA sensor gene methylation status. (**A**) AIM2. (**B**) DDX41. (**C**) DDX60. (**D**) DHX9. (**E**) DHX36. (**F**) HNRNPA2B1. (**G**) IFI16. (**H**) LRRFIP1. (**I**) MRE11. (**J**) POLR3. (**K**) PRKDC. (**L**) TLR9.

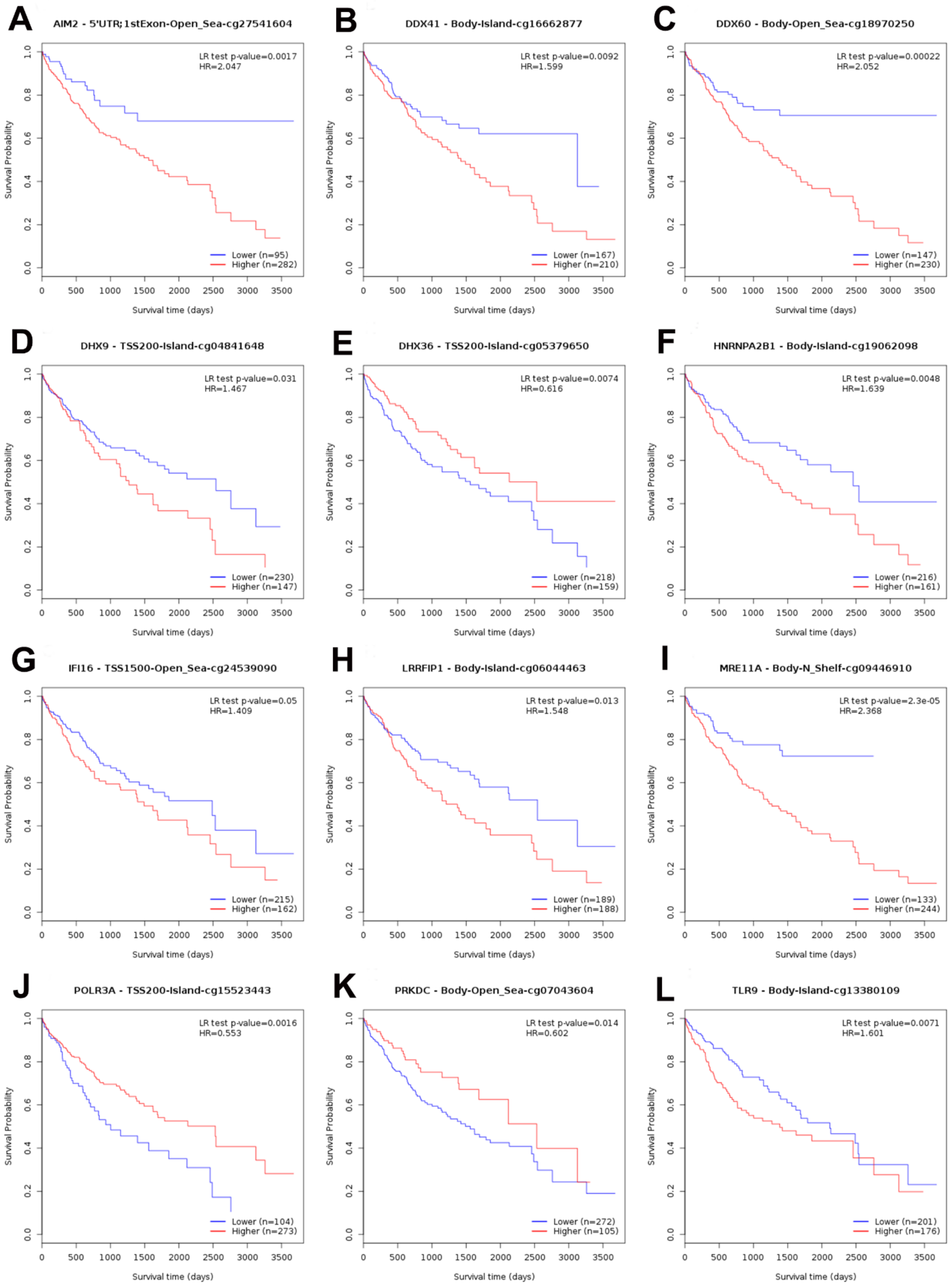

Supplementary Figure 3. Survival analysis of cg sites. (**A**) AIM2. (**B**) DDX41. (**C**) DDX60. (**D**) DHX9. (**E**) DHX36. (**F**) HNRNPA2B1. (**G**) IFI16. (**H**) LRRFIP1. (**I**) MRE11. (**J**) POLR3. (**K**) PRKDC. (**L**) TLR9.

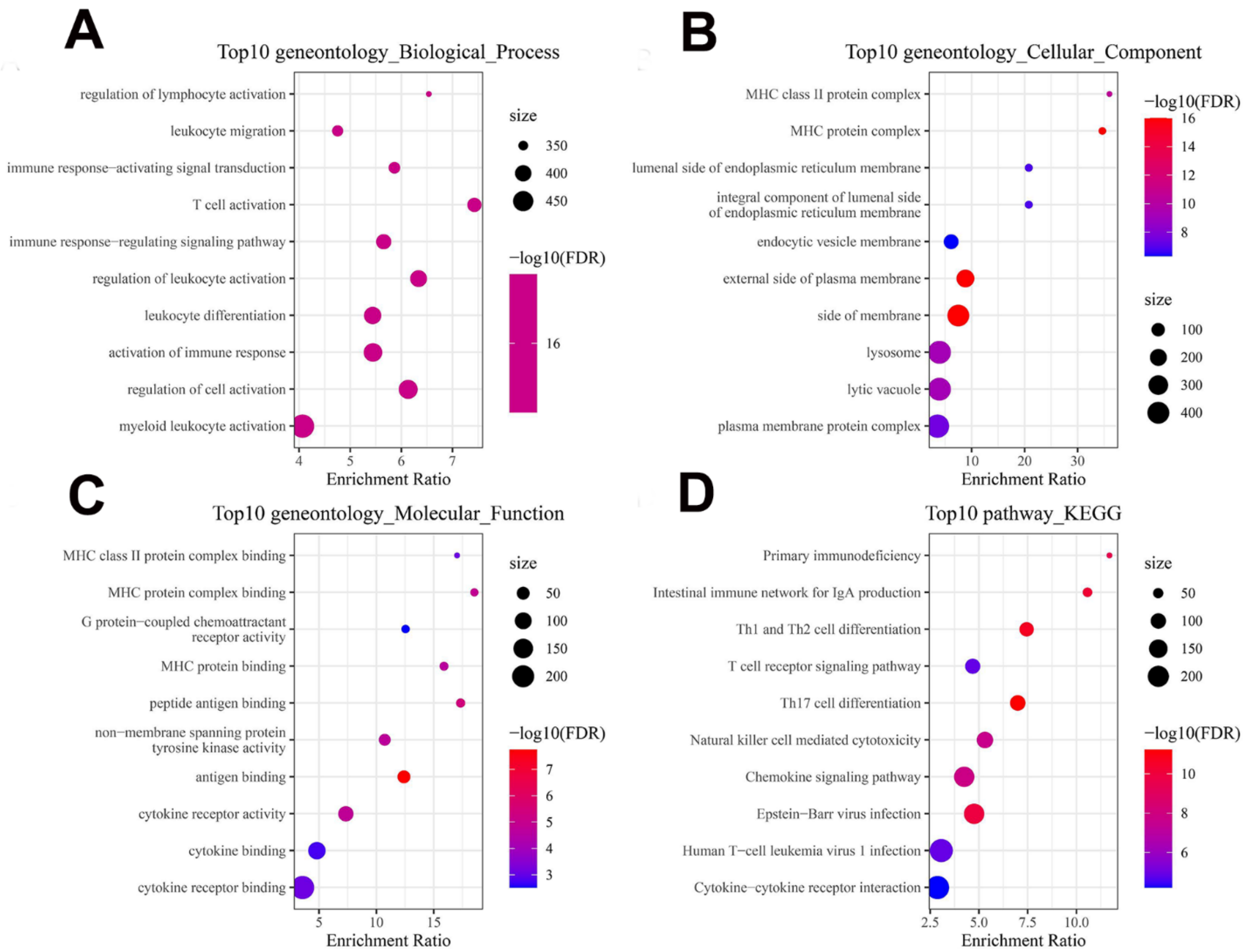

Supplementary Figure 4. DNA sensors-related genes functional enrichment analysis. (**A**) Top 10 geneontology biological process. (**B**) Top 10 geneontology cellular component. (**C**) Top 10 geneontology molecular function. (**D**) Top 10 pathway_KEGG.

A

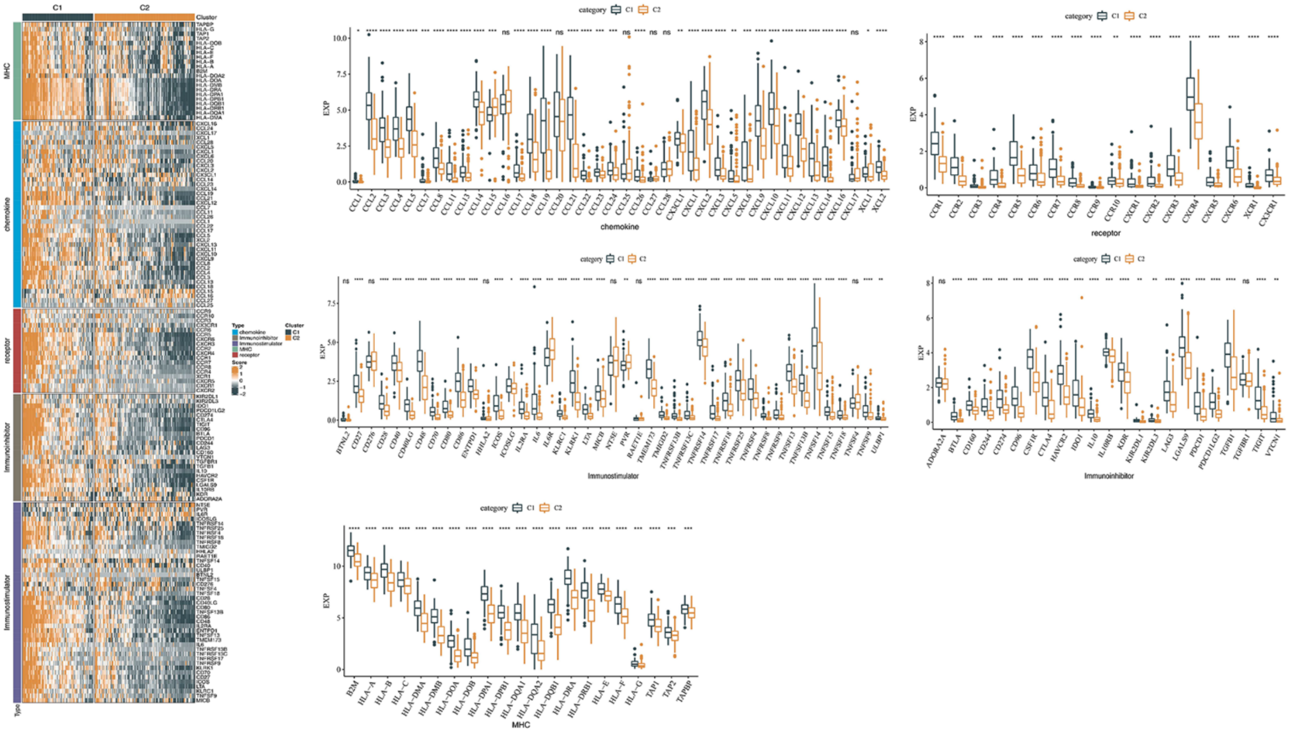

B

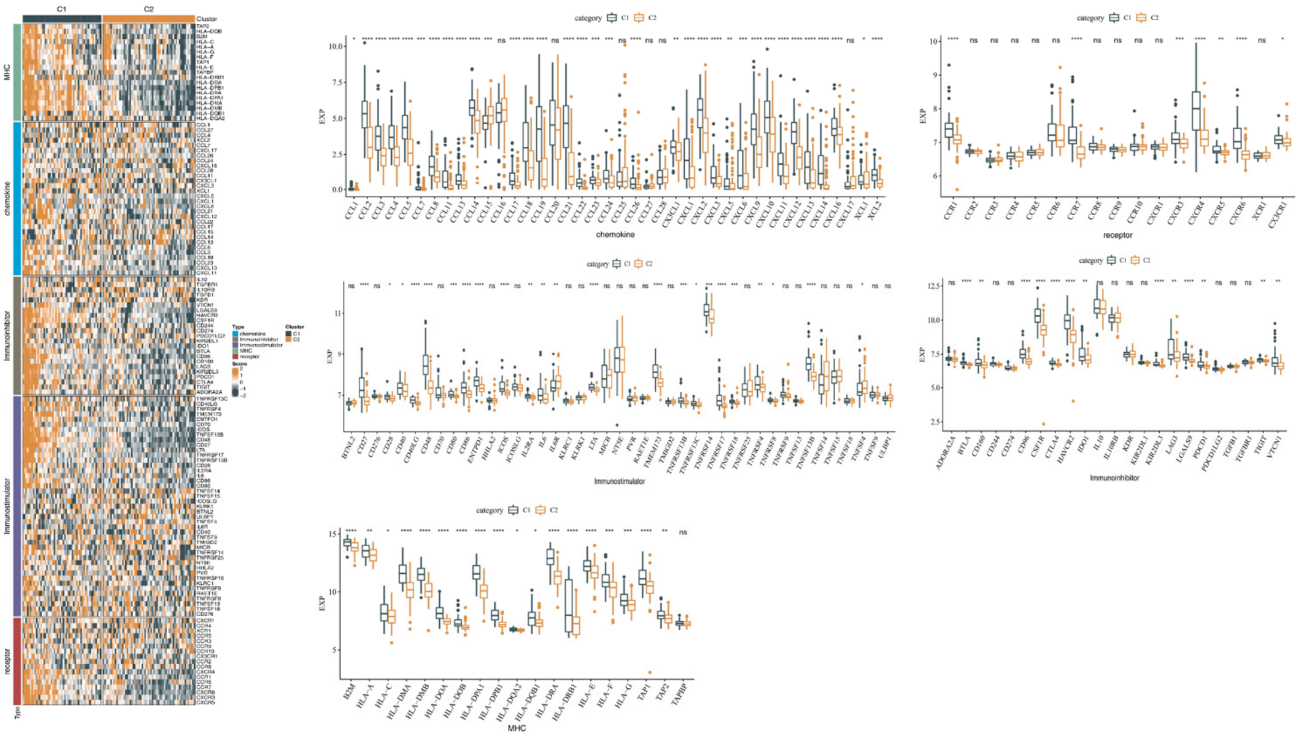

Supplementary Figure 5. Analysis of the immunomodulatory functions of C1 and C2 subtypes. (**A**) Analysis in HCCDB18 dataset. (**B**) Analysis in GSE76427 dataset.
