# Supplementary material for: Molecular subtypes based on DNA sensors predict prognosis and tumor immunophenotype in hepatocellular carcinoma
Source: Aging (Albany NY). 2023 Jul 14;15(14):6798–821. doi: 10.18632/aging.204870 (PMC10415551; doi:10.18632/aging.204870)
Supplement: Supplementary Table 2 [file aging-15-204870-s003.pdf]

SUPPLEMENTARY TABLE

Supplementary Table 2. Primer sequences.

Gene	Forward (5'-3')	Reverse (5'-3')
β-actin	TGGCACCCAGCACAATGAA	CTAAGTCATAGTCCGCCTAGAAGCA
IFI16	TTTCTCTGGGGCAATAGCAGA	CAGAAACGGAACCGCAGGAT
PRKDC	GTTTGATGAGCGGGTGACAG	CTCCTCTTGGGACATGGTGTT
DHX9	GTACGGCCTGGATTCTGCTT	ATCACAGCATCCAAAGGGGG
DDX41	CCGCCTGTACTCATCTTTGC	CAGCACTGACTCATCACACG
cGAS	GGGCGGTTTTGGAGAAGTTG	CGTGCTCATAGTAGCTCCCG
HNRNPA2B1	TGCTCCTCGCAGAGTTGTTT	TTTCTCTCTCCATCGCGGAC
DHX36	AAGGGAACTGCGAAGAAGGT	AGGCATCAGTGAATGTAAAGGT
DDX60	GCGCTAGGTCTCTGTTTCACT	TCCATTCTTGCTGCTGCCTG
